# Ti_3_C_2_T_x_ MXene-Based Fluorescent Aptasensor for Detection of Dimethoate Pesticide

**DOI:** 10.3390/bios14020069

**Published:** 2024-01-29

**Authors:** Zhichao Li, Hongbin Pu, Qingyi Wei

**Affiliations:** 1 School of Food Science and Engineering, South China University of Technology, Guangzhou 510641, China; 202020126004@mail.scut.edu.cn (Z.L.); fehbpu@scut.edu.cn (H.P.); 2 Academy of Contemporary Food Engineering, South China University of Technology, Guangzhou Higher Education Mega Center, Guangzhou 510006, China; 3 Engineering and Technological Research Centre of Guangdong Province on Intelligent Sensing and Process Control of Cold Chain Foods, Guangdong Province Engineering Laboratory for Intelligent Cold Chain Logistics Equipment for Agricultural Products, Guangzhou Higher Education Mega Centre, Guangzhou 510006, China

**Keywords:** carbon quantum dots, aptasensor, dimethoate, MXene, juice, tap water

## Abstract

Dimethoate contaminants in food pose a threat to human health. Rapid and sensitive trace detection methods are required to keep food safe. In this study, a novel fluorescent aptasensor was developed for the sensitive detection of dimethoate based on carbon quantum dots labeled with double-stranded DNA (CQDs−apt−cDNA) and Ti_3_C_2_T_x_ flakes. Under optimal conditions, the aptasensor showed a good linear range of 1 × 10^−9^ to 5 × 10^−5^ M for dimethoate with a coefficient of determination (R^2^) of 0.996. Besides, a low detection limit of 2.18 × 10^−10^ M was obtained. The aptasensor showed high selectivity in interference samples and good reproducibility with an RSD of 3.06% (<5%) for dimethoate detection. Furthermore, the proposed aptasensor was applied to the detection of dimethoate in apple juice and tap water with satisfactory recoveries from 96.2 to 104.4%. Because of these benefits, this aptasensor has the potential and promise for detecting food contaminants in the food industry.

## 1. Introduction

Nowadays, pesticides are commonly used in agricultural products to protect plants and fruits. However, overuse of pesticides can cause an enrichment of residues in the surrounding environment and the food chain, thus posing risks to human health [1,2]. Dimethoate is one of the important organophosphorus pesticides, used to control the growth of insect pests on crops [3,4]. The admissible daily intake for dimethoate is 0.002 mg kg^−1^ body weight day^−1^ [5]. The residues of dimethoate in food may cause serious health problems, including memory loss, anxiety, and depression. Studies proved that heavy exposure to dimethoate can lead to cancer and death [6,7]. In addition, higher toxicity occurs when dimethoate is metabolized to omethoate in animals and plants [8]. Therefore, a simple and sensitive method to detect dimethoate in food and water is vitally important for protecting the ecosystem.

Currently, several methods are used to detect dimethoate including high-performance liquid chromatography (HPLC) [9,10], gas chromatography (GC) with mass spectrometry (MS) [11,12], capillary electrophoresis-mass spectrometry (CE-MS) [13], thin layer chromatography [14], an electrochemical sensor [15,16] and a biosensor [17,18]. These methods are sophisticated, sensitive, and accurate for the determination of dimethoate at low levels. Inevitably, most of them have some disadvantages including the complexity of the operation, time consumption, being labor-intensive, and costly. HPLC has the advantage of high sensitivity and selectivity but requires several complex pre-treatments and is costly. GC is the basic method used for testing organophosphorus pesticides in the environment, although this method also requires several complex sample preprocessing procedures, which can lead to slow speed and poor continuation in practical applications. Surface-enhanced Raman spectroscopy (SERS) is widely used for the detection of dimethoate, a non-destructive technique based on the absorption of target molecules on the nanostructured metallic surfaces (substrate) to enhance the molecular Raman signal [1]. However, SERS is overly dependent on the performance of the substrate, which directly affects its stability and reproducibility [19,20]. Naturally, a better-performing method is required for the detection of dimethoate in the presence of multiple interfering factors.

Fluorescent methods have received much interest from experts in recent years with the advantages of good reproducibility, simplicity, rapid response, and ease of handling [21]. Organic dyes, quantum dots, and fluorescent proteins are used in fluorescent nanomaterials. Carbon quantum dots (CQDs) are a relativity new member of fluorescent carbon nanoparticles with a size below 10 nm and several functional groups (-OH and -COOH) on the surfaces, which are cheap, environmentally friendly, and safe to prepare. Studies showed that CQDs have been broadly applied as excellent fluorescent probes for generating and transmitting fluorescence sensing signals of analytes with the advantages of photochemical stability, long fluorescence lifetime, low toxicity, and high fluorescence quantum production rates [3,15]. Li et al. developed a molecularly imprinted fluorescent sensor based on a competitive reaction for determination of dimethoate with a detection limit of 18 pM [15]. In fluorescence sensing, aptamers are required to bind the analyte to reduce interference from other substances. Generally, aptamers refer to single-stranded synthetic small nucleic acid chains (DNA or RNA) that can selectively bind their targets by shape recognition [16]. Elshafey et al. constructed an electrochemical biosensor based on ds-DNA−GO@GQDs/GCE (graphene oxide and graphene quantum dots) for detecting dimethoate with a detection limit of 1 fM [16]. Compared with antibodies, aptamers are chemically more stable and better suited for site-specific labeling [22]. Consequently, several studies [8,23] have been carried out on aptamer-based fluorescent probes for the detection of pesticides.

MXenes–Ti_3_C_2_T_x_ is an emerging two-dimensional transition metal carbide obtained by selectively etching the atomic layer of Al elements in the layered bulk precursor of Ti_3_AlC_2_, where T_x_ refers to surface-terminating functional groups such as oxygen (O), hydroxyl (-OH), or fluorine (-F). MXenes have large surface areas and can readily adsorb single-stranded DNA (ssDNA) through noncovalent interactions, which enhances the target-induced ssDNA release efficiency [24,25]. Besides, MXenes exhibit broadband absorption in the visible to near-infrared region, as well as long-range electron and energy transfer capabilities, which allow MXenes to significantly quench the fluorescence of the fluorophore, thereby greatly reducing the fluorescence background signals [26,27]. Therefore, MXene could act as a feasible quencher of fluorophores towards the development of sensitive fluorescent aptasensors for effective trace detection of dimethoate.

In this research, a novel fluorescence aptasensor based on the combination of CQDs−apt−cDNA with Ti_3_C_2_T_x_ flakes (CQDs−apt−cDNA/MXene) was developed for the detection of dimethoate. CQDs with a -COOH group were labeled as a dimethoate aptamer with an -NH_2_ group through carbodiimide crosslinker chemistry, and the resulting CQDs−ap−tamer (CQDs−apt) was subsequently bound to complementary DNA (cDNA) to form CQDs−aptamer−cDNA (CQDs−apt−cDNA). In the presence of dimethoate, the dimethoate aptamer in CQDs−apt−cDNA could bind specifically to dimethoate, thereby preventing the aptamer from binding to the double strand of the cDNA. Besides, MXenes provide an ideal fluorescence quenching ability, which makes it possible to quench the fluorescence of CQDs−apt−cDNA by fluorescence resonance energy transfer (FRET). As a result, CQDs-apt-cDNA/MXene assembles with a low fluorescence intensity. When dimethoate was present and incubated with CQDs−apt−cDNA/MXene, and after centrifugation, the dimethoate-bound CQDs were in the supernatant, leading to the restoration of fluorescence, thereby enabling the analysis of dimethoate in trace amounts. Moreover, the effective determination of dimethoate in apple juice and tap water samples further indicated that this aptasensor has great promise for practical applications.

## 2. Experimental Section

### 2.1. Chemicals and Reagents

Citric acid, ethylenediamine, 1-Ethyl-3-(3-dimethyl aminopropyl) carbodiimide (EDC), N-hydroxysuccinimide (NHS), Lithium fluoride (LiF), MAX precursor (Ti_3_AlC_2_), and dimethoate were procured from Shanghai Macklin Biochemical Co., Ltd. (Shanghai, China). Hydrochloric acid (HCl) was obtained from Sinopharm Chemical Reagent Co., Ltd. (Shanghai, China). The dimethoate aptamer and cDNA used in this work were 5′-NH_2_-(CH_2_)_6_-AGC TTG CTG CAG CGA TTC TTG ATC GCC ACA GAG CT-3′ and 5′-AGC TCT GTG GCG ATC AAG AAT CGC TGC AG-3′, respectively, which were obtained from Shanghai Sangon Biotechnology Co., Ltd. (Shanghai, China) [28]. The reagents used were analytically pure and not further purified. Ultrapure water (purity grade, conductivity of 18.2 MΩ·cm) used in all experiments was acquired through the United States Milli-Q water system (Millipore Co., Ltd., Billerica, MA, USA). 

### 2.2. Synthesis and Characterization of CQDs

CQDs were achieved based on our earlier work using a hydrothermal method [29]. In detail, 2.0 g of weighed citric acid was added to 20 mL of ultrapure water. Subsequently, 1.043 mL of ethylenediamine was added to the above mixture. After ultrasonication to dissolve the citric acid evenly, the resolution was heated at 200 °C for 5 min in a Jupiter-b microwave digestion instrument (Sineo Microwave Chemistry Technology Co., Ltd., Shanghai, China), then cooled to room temperature. The faint yellow liquid was first filtered through a microporous membrane (0.22 μm, Jinteng Experiment Equipment Co., Ltd., Tianjin, China) to remove impurities. The CQDs were then dialyzed against ultrapure water for 36 h in a 1000 Da cut-off dialysis bag (Shanghai Yuanye Biotechnology Co., Ltd., Shanghai, China). At last, the purified CQDs solution was kept in a freezer (BCD-539WT, Haier Group, Shandong, China) at 4 °C for later use.

The appearance of CQDs was described by transmission electron microscopy (JEOL Ltd., Tokyo, Japan), which was operated on a JEM-1400 Plus microscope at a voltage of 120 kV. The ultraviolet-visible (UV-vis) of the CQDs was analyzed on a UV-1800 spectrophotometer (Shimadzu Co., Ltd., Kyoto, Japan) using a quartz carrier. The fluorescence emission (EM) spectra of the CQDs were collected through an RF-6000 fluorescence spectrometer (Shimadzu Co., Ltd., Kyoto, Japan) with an excitation (EX) wavelength of 350 nm. The types of surface groups of CQDs were determined by Fourier transform infrared (FT-IR) using a Nicolet-iS50 spectrometer (Thermo-Nicolet Co., Ltd., Waltham, MA, USA).

### 2.3. Preparation and Characterization of CQDs-Apt

Modifying dimethoate aptamer on CQDs was carried out by an acylation reaction [29]. In general, 3 mL of purified CQDs was mixed with 0.02 g of EDC and 0.03 g of NHS, followed by shaking with a digital shaker (MS 3, IKA Inc., Staufen, Germany) for 30 min to reactivate the -COOH groups in the CQDs. Then, 4 μL of 100 μM aptamer was added to the above solution. The mixture was incubated and continuously shaken at 35 °C for 5 h in a water bath (DKZ−2B, Shanghai Yiheng Scientific Instruments Co., Ltd., Shanghai, China). The resulting CQDs-apt solution was dialyzed for 30 h with ultrapure water to clear the unconjugated nanomaterials followed by storing at 4 °C. CQDs-apt was analyzed by the UV-vis absorption and FT-IR spectra collected by a UV-1800 spectrometer and a Nicolet-iS50 spectrometer, respectively.

### 2.4. Preparation and Characterization of Ti_3_C_2_T_x_ Flakes

The preparation of Ti_3_C_2_T_x_ flakes was made based on the literature [30,31] with several adjustments. In general, 2.0 g of LiF was mixed with 40 mL of HCl solution (9.0 M) in a 100 mL plastic bottle under continuous magnetic stirring (DF-101S, Gongyi Yuhua Instrument Co., Ltd., Zhengzhou, China) for 30 min. Subsequently, 2.0 g of Ti_3_AlC_2_ powder was gently introduced into the HF-containing solution and stirred at 25 °C for 24 h. At the end of the reaction, the acidic substance was washed repeatedly with ultrapure water several times and centrifuged at 4000 rpm for 5 min per round until the pH reached 6.0. Afterward, the precipitate was added to 60 mL of ultrapure water and ultrasonically cleaned for 15 min under an N_2_ atmosphere in an ice bath with an ultrasonic cleaner (SB-5200 DTDN, Xinzhi Biotech Co., Ltd., Ningbo, China). The received Ti_3_C_2_T_x_ suspension was centrifuged under 4000 rpm for 15 min. Finally, the suspension of Ti_3_C_2_T_x_ flakes, considered as a monolayer, was collected and used for subsequent study.

The UV-vis absorption spectrum of Ti_3_C_2_T_x_ flakes was obtained on the UV-1800 spectrometer. The structure of Ti_3_AlC_2_ and the d-spacing of Ti_3_C_2_T_x_ flakes were studied using an X-ray diffractometer (XRD) (Empyrean, PANalytical B.V., Almelo, The Netherlands). The elemental composition of Ti_3_C_2_T_x_ flakes was analyzed by X-ray photoelectron spectroscopy (XPS) (Axis Ultra DLD, Kratos Analytical Ltd., Manchester, UK). The field emission scanning electron microscopy (SEM) (Carl Zeiss NTS GmbH, Oberkochen, Germany) was used to observe the shape of Ti_3_AlC_2_ and Ti_3_C_2_T_x_ flakes. The roughness and thickness of Ti_3_C_2_T_x_ flakes were analyzed by an atomic force microscope (AFM) (Horiba France SAS, Villeneuve d’Ascq, France). The morphology and size of Ti_3_C_2_T_x_ flakes were obtained through a JEM-2100F transmission electron microscope (TEM) (JEOL Ltd., Tokyo, Japan). The TEM was equipped with an energy-dispersive X-ray spectrometer (EDS) (Quantax 200, Bruker Co., Ltd., Karlsruhe, Germany) to examine the element composition of Ti_3_C_2_T_x_ flakes. 

### 2.5. Fluorescence Detection of Dimethoate in Standard Solutions

**Scheme 1:**
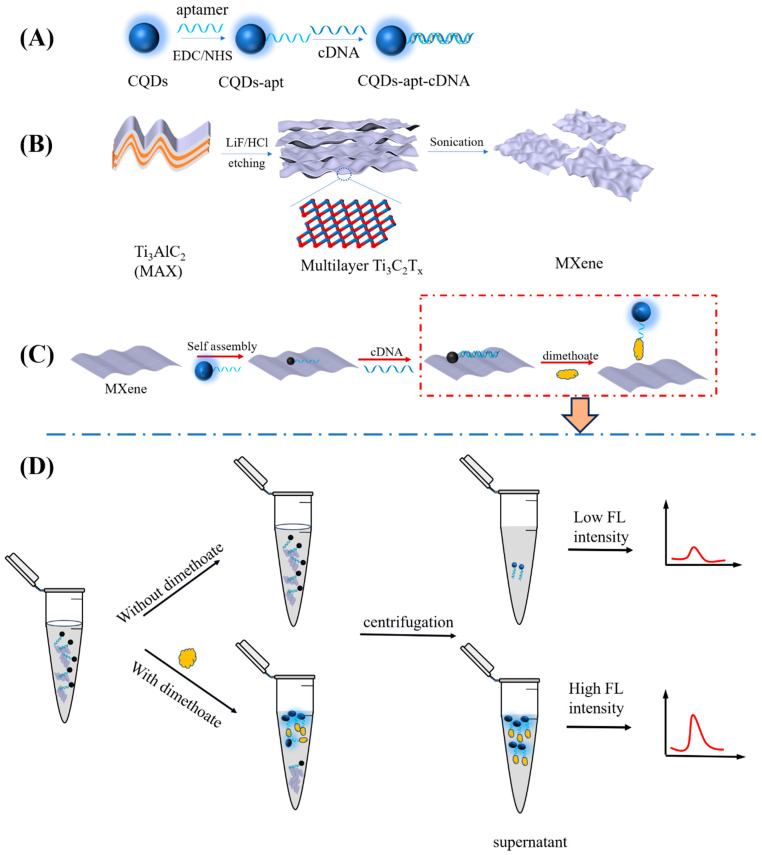
(**A**) Schematic illustration of the synthesis route of CQDs−apt and CQDs−apt−cDNA. (**B**) Schematic illustration of the fabrication of Ti_3_C_2_T_x_ flakes. (**C**) Principle of fluorescence aptasensor for dimethoate determination. (**D**) Schematic illustration of fluorescence aptasensor based on CQDs−apt−cDNA/MXene assemblies for dimethoate detection.

In short, the best fluorescence quenching was achieved by mixing CQDs−apt−cDNA (100 μL) with Ti_3_C_2_T_x_ flakes solution (250 μL) and then adding ultrapure water to a total volume of 500 μL for 25 min. The standard solution of 5 × 10^−4^ M of dimethoate with methanol was accurately prepared and further diluted with methanol to different concentrations step by step. In the detection of dimethoate, as shown in Scheme 1D, ultrapure water (50 μL) was added to CQDs−apt−cDNA (100 μL) and then hybridized with Ti_3_C_2_T_x_ flakes solution (250 μL) for 25 min. Subsequently, 100 μL of different concentrations (10^−9^–10^−4^ M) of dimethoate solutions was added to the mixture and incubated at 37 °C for 60 min. The resulting solution was centrifuged at 4000 rpm for 5 min. The fluorescence spectra of the supernatant were acquired in the emission wavelength range of 400–600 nm, with an excitation wavelength of 350 nm and a maximum fluorescence response at 450 nm.

### 2.6. Detection of Dimethoate in Real Samples

Fresh apples served for testing of dimethoate were bought from local supermarkets (Guangzhou, China). Fresh apple juice samples were prepared according to earlier work [32]. Briefly, the steps for the preparation of fresh apple juice samples included core removal, grinding, homogenization, filtration, sterilization, and filtration. Afterward, the fresh apple juice was centrifuged at 10,000 rpm for 10 min and filtrated using the 0.22 μm microporous membrane to remove the insoluble substance. The apple juice was added with the standard stock solution of dimethoate (1 mM) to obtain different concentrations (0.5, 5, and 50 μM) of the dimethoate apple juice spiked solutions. The different final spiked concentrations of dimethoate (0.1, 1, and 10 µM) in the apple juice samples were detected following the approach of 2.5. Tap water collected through our institutional facility was treated with microporous membrane filtration twice. The rest of the process was the same as for the treatment of the apple juice sample. Finally, the results of the spiked recovery experiments of tap water and apple juice were compared.

### 2.7. Statistical Analysis

Fluorescence intensity and other measurements were repeated three times and expressed as mean ± standard deviation. All experimental data were handled and analyzed through Origin 2021 software (OriginLab Co., Ltd., Northampton, MA, USA). One-way ANOVA and Duncan’s test were performed with SPSS Statistics 26 software (IBM Co., Ltd., Armonk, NY, USA) to analyze acquired data for significant differences (*p* < 0.05). The Nano-Measurer 1.2 software (Department of Chemistry, Fudan University, China) was used to calculate the granularity distribution of CQDs. The fluorescence output intensity became F-F_0_, where F represented the fluorescence intensity in the existence of dimethoate, and F_0_ represented the fluorescence intensity in the disappearance of dimethoate, respectively. 

Ultimately, the limit of detection (*LOD*) and limit of quantification (*LOQ*) of this aptasensor were obtained through the formula:

(1) $$LOD=3S_{h}+Y_{h}$$

(2) LOQ=3.3 LOD

where S_h_ refers to the standard deviation of the fluorescence signal in the blank sample at 450 nm, and Y_h_ represents the average fluorescence intensity of the blank sample.

## 3. Results and Discussion

### 3.1. Mechanism of Fluorescent Aptasensor

The overall process of sample preparation is illustrated in Scheme 1. From Scheme 1A, the preparation of CQDs was accomplished by the microwave-assisted hydrothermal method. The carboxyl group on the surface of CQDs was bound with the dimethoate aptamer containing the amino group via an amide reaction to form CQDs−apt. Subsequently, cDNA was added to form CQDs−apt−cDNA. As illustrated in Scheme 1B, Ti_3_C_2_T_x_ flakes were prepared via in situ hydrogen fluoride etching the precursor of Ti_3_AlC_2_, and then sonicated after intercalation to generate Ti_3_C_2_T_x_ flakes. From Scheme 1C, the dimethoate-binding aptamer was labeled by CQDs (probe), the fluorescence signal of which can be efficiently quenched by Ti_3_C_2_T_x_ flakes through FRET between CQDs and Ti_3_C_2_T_x_. While the hybridization of the probe with its complementary target DNA (cDNA) to form CQDs−apt−cDNA, the weak interaction of CQDs−apt−cDNA and Ti_3_C_2_T_x_ flakes could result in a slight recovery of fluorescence. Owing to the higher rigidity of the double helix compared to the single-stranded form, the adsorption of dsDNA to Ti_3_C_2_T_x_ flakes was typically weaker [33,34]. In the presence of dimethoate, the CQDs−apt−cDNA could combine with dimethoate because of the higher affinity between aptamer and dimethoate. The aptamer-dimethoate with CQDs could detach from the surface of Ti_3_C_2_T_x_ flakes, leading to a decrease in FRET efficiency and recovery of fluorescence. 

### 3.2. Characterization of CQDs and CQDs-Aptamer

**Figure 1:**
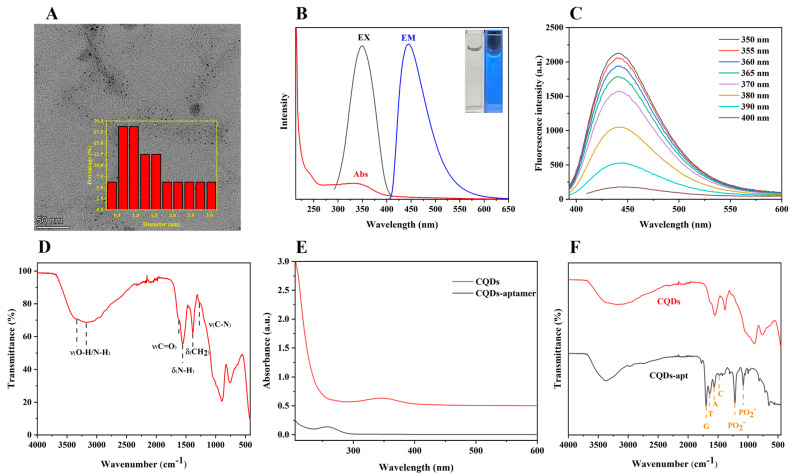
(**A**) TEM image, inset shows the particle size distribution of CQDs; (**B**) UV−vis absorption (Abs), excitation (EX), and emission (EM) spectra of CQDs, and the inset shows the photographs of CQDs in daylight (**left**) and 365 nm ultra−violet light (**right**); (**C**) fluorescence spectra of the CQDs at different excitation wavelengths; (**D**) FT−IR spectra of CQDs; (**E**) UV−vis absorption and (**F**) FT−IR spectra of CQDs and CQDs−apt.

The specific shape and size of CQDs were investigated by TEM measurement. As seen from the TEM image of Figure 1A, CQDs were nearly sphere-like and uniformly dispersed within the ultrapure water. As seen from the inset of particle size distribution in Figure 1A, the average diameter of CQDs was calculated to be 1.2 nm. Subsequently, the optical characteristics of CQDs are shown in Figure 1B. The optimal fluorescence signals of CQDs were achieved at the excitation (EX) and emission (EM) wavelengths of 350 nm and 450 nm, respectively. Additionally, the UV-vis spectrum revealed two broad absorption peaks of the CQDs at 240 and 350 nm, corresponding to the π-π* (C=C) transition and n-π* (C=O) transition, respectively [35,36]. As shown in the inset of Figure 1B, the diluted aqueous solution of CQDs was colorless and transparent in natural light (left), but it could emit bright blue fluorescence under the UV light of 365 nm. These results were consistent with our previous study [37]. The fluorescence emission spectra of CQDs could not change with the change of the excitation wavelength, as shown in Figure 1C. As the excitation wavelength increased from 350 to 400 nm, the emission wavelength remained at 450 nm, without a redshift. Only the emission intensity decreased. The emission intensity of CQDs was strongest at an excitation wavelength of 350 nm. In addition, from Figure 1D, the surface groups of CQDs were explored through the FT-IR spectra. Two obvious wide bands about 3400 and 3250 cm^−1^ belonged to the stretching vibrations of O-H and N-H. The absorption bands around 1620 and 1278 cm^−1^ were ascribed to the C=O and C-N stretching vibrations. In addition, the remaining two absorption bands near 1554, and 1390 cm^−1^ were the deformation vibrations of N-H and -CH_2_. The obtained results indicated that the existence of abundant groups (-NH_2_, -OH, and -COOH) in CQDs conferred good fluorescence and hydrophilicity properties to CQDs.

The dimethoate aptamer labeled on the CQDs assembly was carried out through UV−vis absorption and FT−IR spectroscopic measurements. It could be seen from Figure 1E that the UV spectrum of CQDs−apt exhibited a novel band around 259 nm due to the purines and pyrimidines in the aptamer [38]. The CQDs and CQDs-apt were further compared by FT-IR spectroscopy as depicted in Figure 1F. Different from CQDs, the FT−IR spectra of CQDs−apt showed two novel bands around 1220 and 1080 cm^−1^, which were symmetric as well as asymmetric vibrations for the phosphate group, respectively [29]. In addition, the nitrogenous basebands of guanine (G), thymine (T), adenine (A), and cytosine (C) were placed around 1702, 1643, 1572, and 1490 cm^−1^, respectively, which was consistent with previous studies [39]. These results indicated the successful synthesis of CQDs-apt.

### 3.3. Characterization of Ti_3_C_2_T_x_ Flakes

**Figure 2:**
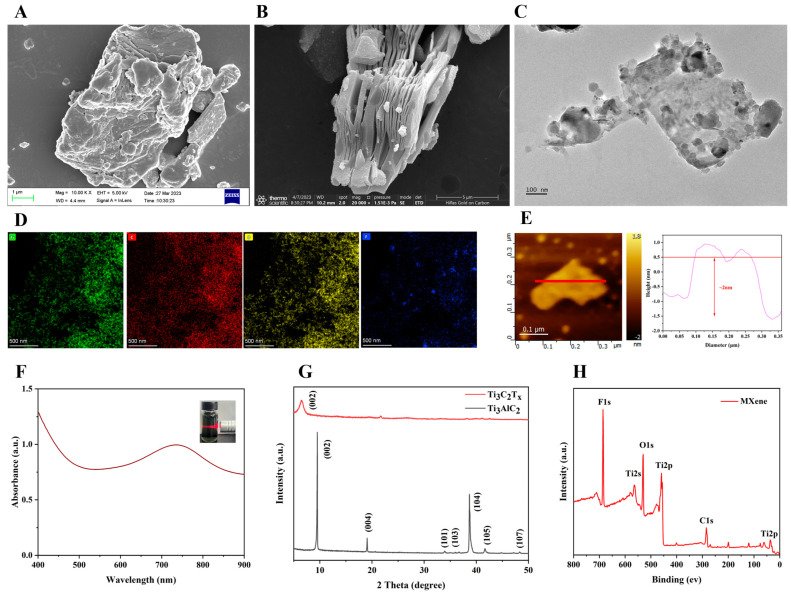
(**A**) SEM image of Ti_3_AlC_2_; (**B**) SEM image of Ti_3_C_2_T_x_ flakes; (**C**) TEM image of Ti_3_C_2_T_x_ flakes; (**D**) EDS mapping images (Ti, C, O, and F) of Ti_3_C_2_T_x_ flakes; (**E**) AFM image of Ti_3_C_2_T_x_ flakes; (**F**) UV-vis absorption of Ti_3_C_2_T_x_ flakes, and the inset shows the photograph of the Ti_3_C_2_T_x_ flakes aqueous solution irradiated with a laser pointer. (**G**) XRD spectra of MAX (black) and Ti_3_C_2_T_x_ flakes (red); and (**H**) XPS survey spectra of MXene.

The morphology characterization of Ti_3_AlC_2_ and Ti_3_C_2_T_x_ flakes was obtained by scanning electron microscopy (SEM), revealing the bulky layered MAX precursor (Figure 2A) and the loosely stacked structure of MXenes (Figure 2B). Subsequently, the HR−TEM image of synthesized Ti_3_C_2_T_x_ flakes was obtained with a flat surface and monodisperse morphology, as depicted in Figure 2C, suggesting that monolayers or several layers of Ti_3_C_2_T_x_ flakes were successfully synthesized. Furthermore, the EDS of Ti_3_C_2_T_x_ flakes displayed in Figure 2D demonstrated that Ti, C, O, and F elements were uniformly distributed in Ti_3_C_2_T_x_ flakes, while the Al elements were not detected [40]. AFM characterizations proved that the average thickness of Ti_3_C_2_T_x_ flakes was approximately 2.0 nm (Figure 2E). 

The UV-vis absorption spectrum of Ti_3_C_2_T_x_ flakes in water was obtained from Figure 2F, which had a broad absorption characteristic from 400 to 900 nm, indicating that Ti_3_C_2_T_x_ flakes could be used as a good quencher for most fluorophores. Meanwhile, when a laser pointer irradiated the dark green aqueous solution of Ti_3_C_2_T_x_ flakes, the Tyndall phenomenon could be seen (inset of Figure 2F), indicating that the obtained Ti_3_C_2_T_x_ flakes were uniformly dispersed as a colloid in the solution [41]. Furthermore, peaks (002) at 9°, (004) at 19°, and (104) at 39° were the three main peaks displayed in the Ti_3_AlC_2_ XRD spectra (black curve in Figure 2G). In the XRD spectra of Ti_3_C_2_T_x_ flakes (red curve in Figure 2G), one of the most intense peaks (104) at 39° of bulk Ti_3_AlC_2_ disappeared, suggesting a good etching effect. In comparison, the main peaks of (002) at 9° and (004) at 19° were shifted at the low-angle area due to the increased interlayer spacing [26]. The XPS pattern further demonstrated the effective formation of Ti_3_C_2_T_x_ flakes. The results of the full XPS spectrum in Figure 2H displayed the components of Ti, C, O, and F. Four different elements, carbon (C 1s, ~285 eV), titanium (Ti 2p, ~459 eV), oxygen (O 1s, ~530 eV), and fluorine (F 1s, ~686 eV) were present in the Ti_3_C_2_T_x_ flakes, with percentages of 14.81%, 12.29%, 33.89% and 39.01%. The results were consistent with the literature description [33]. All of these demonstrated the successful synthesis of Ti_3_C_2_T_x_ flakes.

### 3.4. Feasibility and Optimization of Aptasensor for Dimethoate Detection

**Figure 3:**
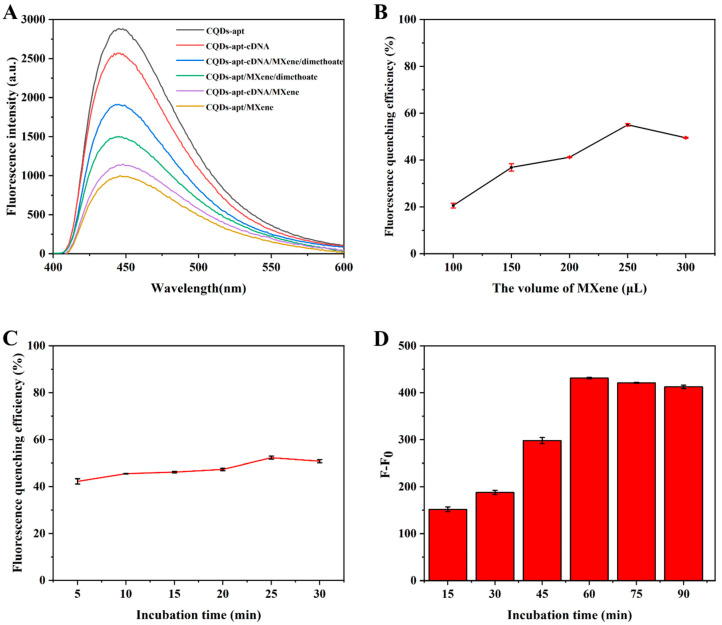
(**A**) Fluorescence spectra of CQDs−apt, CQDs−apt−cDNA, CQDs−apt/MXene, CQDs−apt−cDNA/MXene, CQDs-apt/MXene/dimethoate, and CQDs−apt−cDNA/MXene/dimethoate; (**B**) fluorescence quenching efficiency of CQDs−apt−cDNA combined with different volumes of MXene; (**C**) fluorescence quenching efficiency of CQDs−apt−cDNA under different hybridization times with 250 μL of MXene; and (**D**) relative fluorescence intensities (F−F_0_) of aptasensor when CQDs-apt-cDNA was incubated with dimethoate for different times. F and F_0_ are the fluorescence intensities of the aptasensor with 10^−8^ M dimethoate and without dimethoate.

For testing the viability of this aptasensor, a methanol dimethoate solution (10^−8^ M) was employed in the examination. The fluorescence signal of CQDs−apt exhibited the strongest fluorescence intensity at 450 nm observed in Figure 3A. The cDNA was then introduced into the CQDs−apt through base complementary pairing to form CQDs−apt−cDNA, which was slightly less fluorescent than CQDs−apt. When CQDs−apt was mixed with Ti_3_C_2_T_x_ flakes and separated by centrifugation, the fluorescence intensity of the supernatant dropped significantly, which was ascribed to the selective adsorption of CQDs−apt by Ti_3_C_2_T_x_ flakes through the noncovalent interaction between the phosphate groups of the aptamer and Ti ions on the surface of Ti_3_C_2_T_x_ flakes. The CQDs−apt−cDNA was then mixed with Ti_3_C_2_T_x_ flakes to obtain the CQDs−apt−cDNA/MXene, and the supernatant fluorescence intensity increased slightly after centrifugal separation. When dimethoate was present and incubated with CQDs−apt/MXene or CQDs−apt−cDNA/MXene for 60 min after centrifugation, CQDs−apt from the surface of Ti_3_C_2_T_x_ flakes were released because of the relatively strong specific recognition between the aptamer and dimethoate, leading to higher fluorescence intensity of the supernatant. Compared with CQDs−apt/MXene, CQDs−apt−cDNA/MXene recovered a higher fluorescence intensity than CQDs-apt/MXene in the presence of the same concentration of dimethoate, which could be attributed to the greater stability of the double-strand as well as a weaker interaction between dsDNA and Ti_3_C_2_T_x_ flakes.

It was shown in Figure 3B, that the amount of Ti_3_C_2_T_x_ flakes was improved. The fluorescence quenching efficiency of CQDs−apt−cDNA gradually increased with increasing Ti_3_C_2_T_x_ flakes volume (*p* < 0.05). When the volume of Ti_3_C_2_T_x_ flakes exceeded 250 μL, there was no significant upward trend in fluorescence quenching efficiency. Therefore, 250 μL of Ti_3_C_2_T_x_ flakes were selected for subsequent experiments.

Optimization of hybridization time for CQDs−apt−cDNA and Ti_3_C_2_T_x_ flakes was carried out in Figure 3C. The fluorescence quenching efficiency of the aptasensor could be seen when CQDs−apt−cDNA (100 μL), ultrapure water (150 μL), and MXenes (250 μL) were hybridized for different times (5, 10, 15, 20, 25, and 30 min). The fluorescence quenching efficiency of the aptasensor was highest at 25 min, suggesting that sufficient CQDs−apt−cDNA were loaded on the Ti_3_C_2_T_x_ flakes at this time. Therefore, the optimized hybridization time for CQDs−apt−cDNA and Ti_3_C_2_T_x_ flakes was 25 min.

Different incubation times of the CQDs−apt−cDNA and dimethoate were designed and displayed in Figure 3D. Fluorescence signals (F) were produced when CQDs−apt−cDNA was hybridized with Ti_3_C_2_T_x_ flakes for 25 min followed by incubation with dimethoate for different times (15, 30, 45, 60, 75, and 90 min). The background signals (F_0_) of the aptasensor without dimethoate. It was found that the F−F_0_ increased significantly (*p* < 0.05) with increasing incubation time and attained a maximum value of 60 min. Therefore, 60 min was used as the optimized incubation time for CQDs−apt−cDNA and dimethoate.

### 3.5. Sensitivity Analysis of Aptasensor for Dimethoate Detection

**Figure 4:**
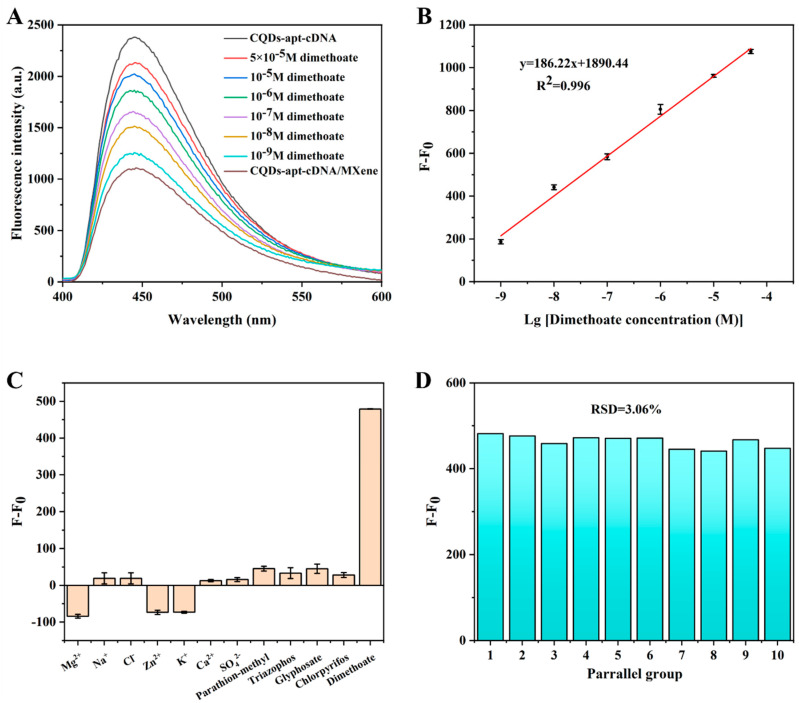
(**A**) Fluorescence spectra of aptasensor with dimethoate concentrations ranging from 1 × 10^−9^ to 5 × 10^−5^ M; (**B**) linear relationship between F−F_0_ and the logarithmic dimethoate concentrations; (**C**) the fluorescent intensities of aptasensor with 10^−5^ M interferences and 10^−8^ M dimethoate; and (**D**) F−F_0_ of 10 parallel samples with 10^−8^ M dimethoate.

Figure 4A showed the fluorescence spectra of the aptasensor in optimal experimental conditions with different concentrations of dimethoate. The sensitivity of the aptasensor was further demonstrated through the fact that the increase in fluorescence intensity was linearly related to the concentration of dimethoate in the concentration range of 1 × 10^−9^ to 5 × 10^−5^ M. The increased fluorescent intensity at 450 nm of the aptasensor was attributed to the binding of more CQDs−apt−cDNA to dimethoate in the supernatant. As shown in Figure 4B, a linear relationship equation for F-F_0_ with the logarithm of dimethoate in the concentration range from 1 × 10^−9^ to 5 × 10^−5^ M was expressed as F−F_0_ = 186.22 × [Log (dimethoate concentration)] + 1890.44 (R^2^ = 0.996). The corresponding LOD and LOQ of dimethoate were calculated as 2.18 × 10^−10^ M and 7.19 × 10^−10^ M. In addition, the findings showed that the aptasensor provided high accuracy and high precision of dimethoate in the standard solutions and could be used for the detection of dimethoate with low concentration. Table 1 compares this work with other reported approaches of detecting dimethoate, further proving that the obtained aptasensor offered a broader detection range, greater reliability, as well as high sensitivity.

### 3.6. Evaluation of Specificity and Reproducibility

Various coexisting substances (parathion-methyl, triazophos, glyphosate, chlorpyrifos) with a structure similar to dimethoate were probed to explore the specificity of this aptasensor. Some ions may interfere with dimethoate (Mg^2+^, Na^+^, Cl^−^, Zn^2+^, K^+^, Ca^2+^, SO_4_^2−^) present in water samples were also chosen as interfering substances for the experiment. We found that the coexisting substances at a concentration of 10^−5^ M did not cause changes in the fluorescence intensity, as shown in Figure 4C. In addition, only a concentration of 10^−8^ M of dimethoate resulted in a significant enhancement of fluorescence intensity (*p* < 0.05). Our findings were consistent with the previously expressed study [42,43], which also proved that the fluorescence intensity of the chosen interfering substances was lower compared to the fluorescence intensity of dimethoate, indicating that the proposed aptasensor was able to specifically detect dimethoate. Furthermore, in reproducibility experiments, 10 parallel samples were carried out using a concentration of 10^−8^ M of dimethoate. Figure 4D showed that the relative standard deviation (RSD) of F−F_0_ was 3.06%, which demonstrated that the aptasensor had good repeatability in detecting dimethoate.

**Table 1:** Comparison of different sensors for dimethoate detection.

Type of Sensor	Detection Range	LOD	Applicability	Reference
Raman based on Ag, CuO and Ag-Cu NPs	3–20 μM	3 μM	Water	[20]
SERS based on AgNPs	0.5–10 μM	0.5 μM	Olive leaves	[19]
Colorimetric sensor based on AuNPs	1–40 nM	6.2 nM	Tap water, green tea, apple juice	[44]
Colorimetric sensor based on MIP-CoZn ZIF	0.02–1.2 μM	5.6 nM	Fruit, wastewater	[7]
Fluorescent sensor based on animal waste biomass carbon dots	0.15–5 μM	0.064 μM	Environment water	[3]
Electrochemical sensor based on MIP-glassy carbon electrode	0.1–1 nM	Not mentioned	Wheat flour	[45]
MIP fluorescent sensor based on FRET	0.6–34 nM	18 pM	Vegetables	[15]
Fluorescent sensor based on AgNPs/OxMWCNTs	0.044–1.528 μM	0.013 μM	Lake water, orange	[46]
Fluorescent biosensor based on CQDs and MXene	0.001–50 μM	0.218 nM	Apple juice, tap water	This work

Note: NPs: nanoparticles; MIP: molecularly imprinted polymer; ZIF: Zeolitic imidazole framework; oxMWCNTs: oxidized multiwalled carbon nanotubes.

### 3.7. Application in Real Samples

Taking into account the possible presence of dimethoate in food, the standard addition method was carried out to detect dimethoate in apple juice and tap water samples. Under a brief pre-processing and combination with dimethoate, samples (apple juice and tap water) were bound to CQDs-apt-cDNA/MXene to obtain dimethoate spiked solutions at concentrations of 0.1, 1, and 10 µM, respectively. After centrifugal separation, the fluorescence signal of the supernatant was obtained. As displayed in Table 2, the findings showed excellent recoveries ranging from 96.2% to 104.4% with an RSD of 2.42–3.89% (<5%), suggesting the great accuracy of this aptasensor.

## 4. Conclusions

During this work, a novel fluorescence aptasensor for dimethoate detection was developed by utilizing CQDs-apt-cDNA as well as Ti_3_C_2_T_x_ flakes. The fluorescence of CQDs-apt-cDNA was quenched by Ti_3_C_2_T_x_ flakes via the FRET mechanism and restored by the addition of dimethoate. The specific recognition of aptamer and the great optical performance of CQDs guaranteed high sensitivity with specificity of the obtained aptasensor. Additionally, the linear range from 1 × 10^−9^ to 5 × 10^−5^ M and the LOD of 2.18 × 10^−10^ M were obtained. Remarkably, the aptasensor was capable of detecting dimethoate in apple juice and water samples with a simple pretreatment. Finally, owing to its excellent performance, this aptasensor offered a bright opportunity and promising approach for detecting other hazardous substances by fabricating the fluorescence aptasensor. However, due to the introduction of the aptamer, the incubation time for CQDs-apt-cDNA and dimethoate was relatively long (60 min). Therefore, nowadays this aptasensor is not suitable to perform real-time or in situ detection.

## Figures and Tables

**Table 2:** Recoveries of dimethoate in real samples based on this aptasensor.

Samples	Dimethoate Add (μM)	Detected Concentration (μM)	Recovery (%)	RSD (%, *n* = 3)
Apple juice	0.1	0.099	99.6	2.52
	1	1.012	101.2	3.18
	10	10.436	104.4	2.84
Tap water	0.1	0.097	97.4	2.42
	1	0.991	99.1	3.60
	10	9.616	96.2	3.89

## Data Availability

Data are contained within the article.
